# *PPBP* gene as a biomarker for coronary heart disease risk in postmenopausal Thai women

**DOI:** 10.7717/peerj.13615

**Published:** 2022-06-17

**Authors:** Chayasin Mansanguan, Yaowapa Maneerat

**Affiliations:** 1 Department of Clinical Tropical Medicine, Faculty of Tropical Medicine, Mahidol University, Bangkok, Thailand; 2 Department of Tropical Pathology, Faculty of Tropical Medicine, Mahidol University, Bangkok, Thailand

**Keywords:** PPBP, Biomarker, Coronary heart disease, Postmenopause, Female

## Abstract

**Background:**

Estrogen is an important ovarian hormone with anti-atherogenic and cardioprotective effects. Postmenopausal women have lower estrogen levels, associated with significantly higher risks of coronary heart disease (CHD) and CHD-related death. Effective biomarkers for the diagnosis, prediction, and treatment of CHD are needed to address this problem and thus reduce the mortality due to CHD in postmenopausal women. We recently reported that the *PPBP* and *DEFA1/DEFA3* genes may be feasible synergistic biomarkers for CHD risk in Thai men with hyperlipidemia. The *PPBP* gene encodes pro-platelet basic protein (PPBP) from activated platelets, and *DEFA1/DEFA3* encodes human neutrophil peptides (HNP) 1–3, mainly produced by activated neutrophils. Both platelets and neutrophils are involved in chronic inflammation during the development of atherogenesis and CHD. This study investigated the potential roles of *PPBP* and *DEFA1/DEFA3* and their proteins as biomarkers for CHD risk in postmenopausal Thai women.

**Methods:**

This cross-sectional study enrolled 90 postmenopausal Thai women, including 12 healthy controls (N), 18 patients with hyperlipidemia (H), and 21 patients diagnosed with CHD. The remaining 39 women were receiving cholesterol-lowering drugs for hyperlipidemia (HD) were excluded from the study. All CHD patients underwent coronary bypass grafting or coronary angioplasty. *PPBP* and *DEFA1/DEFA3* mRNA expression levels in peripheral blood mononuclear cells isolated from heparinized blood were determined by quantitative reverse-transcription polymerase chain reaction. Levels of PPBP and HNP-1–3 proteins in corresponding plasma samples were assessed by enzyme-linked immunosorbent assay. Differences in parameters were compared among groups and correlations between parameters and clinical manifestations were analyzed.

**Results:**

PPBP mRNA and protein levels were significantly increased in the CHD group compared with the N and H groups. In contrast, *DEFA1/DEFA3* mRNA and HNP-1–3 protein levels did not differ significantly among the groups. None of the levels were associated with any of the clinical parameters analyzed in this study.

**Conclusion:**

The results indicate that gene and protein expression levels of PPBP, but not *DEFA1/DEFA3*, and HNP-1–3, may be feasible biomarkers for assessing CHD risk in postmenopausal Thai women with hyperlipidemia.

## Introduction

Atherosclerosis involves chronic progressive inflammation of the blood vessel wall, and is characterized by fatty plaque development or the accumulation of lipids and fibrous elements in large and medium-sized arterial walls. It is the most common underlying cause of cardiovascular disease (CVD), which is in turn the leading cause of death in the developed world and an important cause of morbidity worldwide. Atherogenesis and coronary heart disease (CHD) involve a long preclinical process. Complicated risk factors for CHD have been identified in both women and men without familial hypercholesterolemia, including behavioral, dietary, and lifestyle factors such as smoking, physical activity, dietary fat intake, exogenous infections, changes in endogenous blood constituents such as lipid and lipoprotein particles, inflammation and coagulation proteins, intermediate metabolites, and oxidant markers of stress, obesity, hypertension, and diabetes mellitus (Anand et al., 2008; Gonzalez-Jaramillo et al., 2022; Sucato et al., 2022). Sex is also an important factor affecting the development of CVD. The ovarian hormone estrogen is an anti-atherogenic and cardioprotective factor, and premenopausal women with normal estrogen levels are thus relatively protected against coronary artery disease and CHD compared with age-matched men. However, this sex difference narrows after the menopause. Postmenopausal women (usually 44–55 years old, average 50 years), with 12 consecutive months without menstruation, have lower levels of estrogen because of cessation of ovary function, as the main source of estrogen. Postmenopausal women consequently have a significantly higher risk of CHD than premenopausal women. These findings have been supported by treatment with estrogen or estrogen analogs in postmenopausal women and animal models (reviewed in Garcia et al. (2016), Nie et al. (2022)). Moreover, hyperlipidemia is the major population-adjusted risk factor for CVD in women, accounting for approximately 47.1% of the known risk for CVD (reviewed in Garcia et al. (2016)). Effective biomarkers for the diagnosis, prediction, and follow-up of CHD are therefore needed to reduce CHD-related mortality in postmenopausal women with hyperlipidemia.

Previous studies in humans and animal models demonstrated a link between dyslipidemia and atherogenesis, and clarified the roles of chronic inflammation coupled with dyslipidemia in plaque formation (Fan & Watanabe, 2003; Libby, 2012; Weber & Noels, 2011). Atherogenesis is initially characterized by inflammatory cell recruitment combined with pro-inflammatory cytokine expression (Libby, 2012). Inflammatory pathways also contribute to the development of thrombosis, as a serious, late complication of atherosclerosis leading to myocardial infarction and CHD, and associated with an increased risk of sudden death (Weber & Noels, 2011).

Previous studies of the pathogenesis of atherosclerosis revealed that the progression of early-stage (fatty streak) atherosclerosis to more complicated lesions involved chronic inflammation. This develops from interplays among plasma lipoproteins, activated immune cells such as monocytes, macrophages, T cells, and B cells, cellular components of the arterial wall, including endothelial cells and smooth muscle cells, and the extracellular matrix (Libby, 2012). Notably, neutrophils have also demonstrated a notable innate immunity role in atherogenesis in humans (Dorweiler et al., 2008) and animal models, including mice (van Leeuwen et al., 2008) and pigs (Kougias et al., 2006). Neutrophils were associated with plaque rupture and erosion of human lesions, and in thrombi in patients with acute coronary syndrome (Naruko et al., 2002). In addition, the number of neutrophils in the blood circulation and the levels of neutrophil-produced elastase and myeloperoxidase are correlated with CVD, atherosclerosis (Naruko et al., 2002; Ovbiagele et al., 2007), and myocardial infarction (Kawaguchi et al., 1996; Wang et al., 2022).

During inflammation, activated neutrophils produce and release large amounts of intracellular proteins into the extracellular environment by degranulation, leakage during phagosome formation, and cell death. The highly homologous human neutrophil peptides (HNPs)-1, -2, and -3 (HNP-1-3) (also known as α-defensin), comprise a cysteine-rich positively-charged polypeptide that accounts for more than half of the total protein composition within neutrophilic azurophilic granules (Ganz & Lehrer, 1994). The α-defensin genes (*DEFA1/DEFA3*) encode HNP-1-3 (Aldred, Hollox & Armour, 2005; Linzmeier & Ganz, 2005), which in turn play roles in endothelial cell dysfunction during early atherogenesis. HNP levels were also shown to be markedly elevated in inflammation in sepsis and in acute coronary vascular disorders (Hansson, 2005). We accordingly found that HNP-1–3 expression levels were associated with CHD development in men with hyperlipidemia (Maneerat et al., 2016, 2017).

Platelets are anuclear cellular fragments derived from megakaryocytes in the bone marrow, which play an important role in the hemostatic system (Junt et al., 2007). In addition to the various inflammatory cells mentioned above, platelets and platelet-derived factors have long been implicated in atherogenesis and the development of CVD. Platelets are also involved in thrombus formation in response to vascular injury, and can affect the coronary, cerebral, and peripheral circulations (Meadows & Bhatt, 2007). After activation, platelet α-granules rapidly release chemokines, which play an important role in atherogenesis. Most platelet-derived chemokines induce the recruitment of hematopoietic cells to the vascular wall, thus fostering processes such as plaque formation, atherosclerosis, and thrombosis. Platelets are also involved in the proliferation, differentiation, and degranulation of various cell types (Gleissner, von Hundelshausen & Ley, 2008).

Pro-platelet basic protein (PPBP), or chemokine (C-X-C motif) ligand 7 (CXCL7), is a small cytokine released by activated platelets and involved in the response to vascular injury (Stankiewicz et al., 2014). PPBP enhances various processes including mitogenesis, extracellular matrix and plasminogen activator synthesis, and glucose metabolism (Hristov et al., 2007).

We previously conducted cross-sectional studies to evaluate predictive biomarkers of CHD in Thai men with non-familial hyperlipidemia. We found that *PPBP* and *DEFA1/DEFA3* were potentially correlated with CHD development, and also showed promise as inflammatory markers to help predict the risk of CHD in hyperlipidemic Thai patients (Maneerat et al., 2017).

The present study aimed to determine the values of the *PPBP* and *DEFA1/DEFA3* genes and their encoded proteins as biomarkers of CHD risk in postmenopausal Thai women with hyperlipidemia.

## Materials and Methods

### Materials

Dulbecco’s phosphate-buffered saline (D-PBS) and TRIzol® reagent were purchased from Invitrogen (Carlsbad, CA, USA). Luna Universal One-Step Reaction mix (2×) was purchased from BioRad Laboratories Inc. (Hercules, CA, USA). Human CXCL7/PBP matched antibody pair kit (ab219537) and enzyme-linked immunosorbent assay (ELISA) accessory pack (ab210905) were purchased from Abcam (Cambridge, UK). Human HNP-1–3 ELISA reagents were purchased from Hycult Biotech (Uden, the Netherlands). All other reagents were from Sigma-Aldrich (St. Louis, MO, USA).

### Study design and patient population

**Figure 1:**
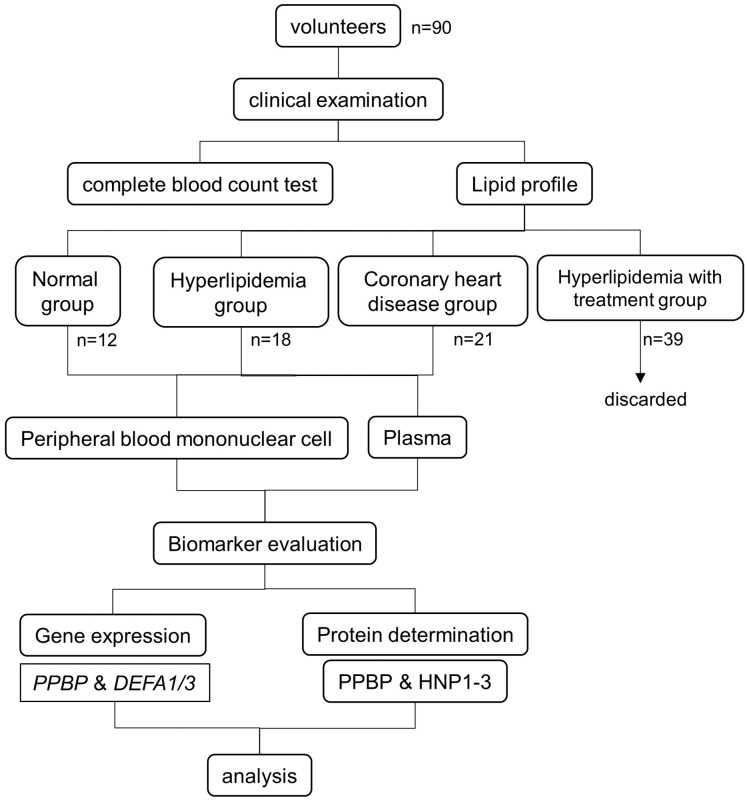
Experimental design and study population.

The patient flow and experimental design are summarized in Fig. 1. The study was conducted at the Faculty of Tropical Medicine, Mahidol University, Thailand. The study was approved by the Ethical Committees of the Faculty of Tropical Medicine (MUTM 2020-007-01, MUTM 2020-007-02) and Bhumibol Adulyadej Hospital (No. 43/64). All participants were informed of the study objectives and completed an informed consent form before enrollment. The sample size calculation from two independent means formula (Rosner, 2000) was made by assuming baseline of mean and standard deviation of all parameters obtained from our previous study (Maneerat et al., 2017). The calculated sample size of hyperlipidemia and coronary heart disease groups are 20 and 35 cases, respectively.

### Patients

All the participants were unrelated postmenopausal women born to Thai parents. Seventy-eight patients were diagnosed, classified, and treated by a specialist (CM) at the Hospital for Tropical Diseases, Faculty of Tropical Medicine, and Bhumibol Adulyadej Hospital, Thailand. They were classified into three groups based on their clinical manifestations, according to the American College of Cardiology/American Heart Association criteria (2013) (Goff et al., 2014): 18 patients had high cholesterol levels (H group) (total cholesterol (TC), low-density lipoprotein (LDL), and high-density lipoprotein (HDL)), 39 patients had high cholesterol levels and received cholesterol-lowering drugs (HD group), and 21 patients were diagnosed with CHD (CHD group). No patients in the H and HD groups had any evidence of vital organ dysfunction. We also enrolled 12 healthy controls with no infections, underlying disease, or CVD risk factors (N group). No H patients or controls received any cholesterol- or blood pressure-lowering medication.

### Blood sample collection and methods

Whole blood samples (10 ml) were collected once from healthy controls and patients into EDTA blood vacutainer tubes with serum clot activator before hyperlipidemia treatment (except HD group) or coronary bypass grafting. Plasma (2 ml) was immediately collected by centrifugation of whole blood samples and prepared for lipid analysis, and kept at −70 °C for the detection of plasma PPBP and HNP-1–3. Serum levels of lipids (TC, triglycerides (TG), HDL, and LDL) were measured.

Packed blood cells were resuspended in D-PBS (Carlsbad, CA, USA) and used to isolate mononuclear cells. Approximately 2 × 10^6^ peripheral blood mononuclear cells (PBMCs) in TRIzol (Invitrogen, Carlsbad, CA, USA) were kept at −70 °C for determination of *PPBP* and *DEFA1/DEFA3* mRNA expression levels by quantitative reverse transcription-polymerase chain reaction (RT-qPCR) analysis, using specific primers. Gene expression was expressed as fold-change relative to the housekeeping β-actin gene (*ACTB*) (Kotepui et al., 2012; Maneerat et al., 2017).

### Lipid test

Lipid markers including TC, TG, LDL, and HDL were analyzed enzymatically using kits (Randox Laboratories Ltd., Crumlin, UK) and a biochemistry analyzer (Architect CI 16200; Abbott Laboratories, Abbott Park, IL, USA).

### Investigation of *DEFA1/3* and *PPBP* mRNA expression by RT-qPCR

RT-qPCR was performed in duplicate. Each 10-µl RT-qPCR reaction contained 5 µl of Luna Universal One-Step Reaction mix (2×) (BioRad Laboratories, Hercules, CA, USA) mixed with 10 ng of RNA. Total RNA was isolated from 2 × 10^6^ PBMCs using TRIzol (Invitrogen, Carlsbad, CA, USA) and 0.4 µm of each set of forward and reverse primers. The primers were designed based on the *DEFA1/DEFA3* genes (GenBank accession numbers NM_005217.3) (forward: 5′-TCCTTGCTGCCATTCTCCTG-3′ and reverse: 5′-TGCACGCTGGTATTCTGCAA-3′) (Li et al., 2014) and *PPBP* gene (GenBank accession numbers NM_002704.3) (forward: 5′-TTGTAGGCAGCAACTCACCC-3′ and reverse: 5′-TGCAAGGCATGAAGTGGTCT-3′ (Yeo et al., 2016). The expected PCR product sizes were 204 bp and 135 bp, respectively. The RT-qPCR reaction was run in a Bio-Rad CFX96 Real-time system (BioRad Laboratories, Hercules, CA, USA). The RT-qPCR conditions were as follows: 55 °C for 10 min and 95 °C for 1 min, followed by 40 cycles of denaturation at 95 °C for 10 s, extension at 60 °C for 30 s (+plate read), and melting curve analysis at 65 °C for 5 min. The expected PCR product size was 204 bp. *ACTB* primers (forward: 5′-TCACCCACACTGTGCCCATCTACGA-3′ and reverse: 5′-CAGCGGAACCGCTCATTGCCAATGG-3′) (Heid et al., 1996; Kotepui et al., 2012) were used to normalize the relative expression levels of *DEFA1/DEFA3* and *PPBP*. The 2^−(ΔΔCt)^ method was used to quantify relative expression levels (Kotepui et al., 2012; Maneerat et al., 2017).

### Determination of protein levels by ELISA

The plasma kept at −70 °C was thawed to room temperature (RT) and then centrifuged at 2,500 × *g* for 5 min. PPBP and HNP-1−3 protein levels were determined in the clear plasma.

### Determination of plasma HNP-1–3 levels

Plasma HNP-1–3 concentrations were measured in triplicate using ELISA kits (Hycult Biotech, Uden, Netherlands), according to the manufacturer’s instructions. Briefly, 100 μl of each plasma from the three groups (N = 12, H = 18, and CHD = 21) was diluted 20-fold in sample dilution buffer, and standards were prepared at concentrations of 10,000–156 pg/ml. The diluted samples and two-fold serial diluted standard were transferred to microtiter wells coated with captured antibody to HNP-1–3 and incubated for 60 min at RT. Each well was then washed to remove unbound material and biotinylated tracer antibody to HNP-1–3 (100 μl) was added, followed by incubation for 60 min at RT. After washing to remove unbound material, streptavidin-peroxidase conjugate was added (100 μl/well) and incubated for 60 min at RT. Bound enzyme was detected by adding 100 μl of tetramethylbenzidine substrate to each well, and reactions were stopped by adding 100 μl of stop solution. The optical density was determined at 450 nm using an Ao Microplate Reader (Azure Biosystems, Dublin, CA, USA). Plasma levels of HNP-1–3 were calculated from the HNP-1–3 standard curves (Maneerat et al., 2016).

### Determination of plasma PPBP levels

Plasma PPBP levels were measured in triplicate using a Human CXCL7/PBP matched antibody pair kit (ab219537) and ELISA accessory pack (ab210905) (Abcam, Cambridge, UK), according to the manufacturer’s instructions as previously described (Maneerat et al., 2016). Briefly, 100 μl of plasma from all groups, as above, were diluted 5,000-fold in sample dilution buffer, and standards at concentrations of 1,000–15.6 pg/ml were transferred to microtiter wells coated with 2 μg/ml of the specific captured antibody and incubated for 2 h at RT. Each well was then washed to remove unbound material and 0.5 μg/ml of biotinylated detector antibody was added, followed by incubation for 1 h at RT. After washing unbound material, 0.02 μg/ml of streptavidin-horseradish peroxidase conjugate was added and incubated for 1 h at RT. Bound enzyme was detected by adding 100 μl of tetramethylbenzidine substrate to each well, and reactions were stopped by adding 100 μl of stop solution. The optical density was measured at 450 nm using an Ao Microplate Reader (Azure Biosystems, Dublin, CA, USA). Plasma levels of PPBP were calculated from the PPBP standard curves (Maneerat et al., 2017).

### Statistical analysis

All data were determined for normality using the kolmogorov–Smirnov test. Most were non-normally distributed variables. Clinical data, plasma levels of PPBP and HNP-1–3 were reported as median (minimum, maximum). mRNA expression was presented as fold-change relative to mRNA in healthy controls. Parameters were compared between two and more than two groups by Mann–Whitney test and Kruskal–Wallis test, respectively. Correlations between parameters and clinical data were analyzed by the Spearman rank correlation test. The α level was set at <0.05 with a 95% confidence interval. All statistical analyses were performed using SPSS version 18 (SPSS, Chicago, IL, USA).

## Results

### Characteristics and clinical manifestations of controls and patient groups

The general data and clinical manifestations of the controls and patients are shown in Table 1. Patients were classified based on their clinical manifestations according to the American College of Cardiology/American Heart Association criteria (2013) (Goff et al., 2014). Our criteria for patient selection was previously described (Maneerat et al., 2016). Especially, the H group had high cholesterol levels (TC, LDL, HDL), but no evidence of vital organ dysfunction. Before inclusion, we confirmed that participants in the N and H groups had not received any cholesterol- or blood pressure-lowering medication. CHD patients were about to undergo coronary bypass grafting. All CHD patients had been treated with statins since they were diagnosed and after surgery. None of the N or H patients had any infections or underlying diseases. As shown in Table 1, there was no significant difference in age between the N and H groups (*p* > 0.05), but patients in the CHD group were significantly older than the other groups (*p* < 0.0001). The differential complete blood counts (CBC) were similar in all three groups (*p* > 0.05) except number of platelets in H were significantly higher than CHD groups (*p* = 0.0115), and monocyte in CHD were significantly higher than the other groups (*p* < 0.0001). The TC (*p* < 0.0001), and LDL levels (*p* = 0.0003, and *p* < 0.0001) were significantly higher in the H compared with the N and CHD groups. The HD group included participants with hyperlipidemia who were taking lipid-lowering drugs daily. These women showed significant differences in most lipid parameters, including TC (*p* < 0.0001), TG (*p* = 0.0048), and LDL (*p* < 0.0001), compared with the H group. The significant difference in CBC between the H and HD groups included differences in white blood cell count (*p* = 0.0146), RBC (*p* = 0.0280), hematocrit (*p* = 0.0004), platelets (*p* = 0.0292), and neutrophils (*p* = 0.0147) (Table S1). These findings indicated that the clinical manifestations in the HD group were significantly affected by lipid-lowering treatment. The HD group was therefore excluded from subsequent analyses.

**Table 1:** General description and clinical manifestations of the study groups.

Variables		N	H	CHD	*p*-value* in 3 group	*p*-value**		
		*(n = 12)*	*(n = 18)*	*(n = 21)*		N *vs*. H	N *vs*. CHD	H *vs*. CHD
Age (year)		56 (42–63)	57 (47–72)	69 (46–88)	<0.0001	0.2324	<0.0001	<0.0001
**Complete blood count**								
	WBC (10^3^/ul)	6 (4.5–8.6)	7 (4.1–8.1)	6 (4.7–9.6)	ns			
	RBC (10^3^/ul)	4.1 (2.4–5.2)	4.4 (2.9–6.0)	4.4 (2.8–5.4)	ns			
	Hb (g/dL)	12.1 (7.3–29.8)	12.8 (0.8–14.8)	12.9 (8–14.7)	ns			
	HCT (%)	35 (21.2–81.3)	38.2 (25.4–44.6)	39.4 (24.8–46.2)	ns			
	PLT (10^3^/ul)	273 (177–406)	289.5 (206–415)	229.5 (155–388)	0.0496	0.3517	0.3118	0.0115
	NEUT (%)	51.7 (35–67)	52.4 (33–60.9)	54.2 (36–77)	ns			
	LYMPH (%)	37.9 (20–59)	38.7 (3.2–54)	31.5 (10–48)	ns			
	MONO (%)	5.8 (4–9.3)	5 (3.3–6.2)	6.6 (3.7–12)	0.0006	0.1359	0.0404	<0.0001
	EO (%)	2.5 (1–7.3)	2.1 (1–5)	2.4 (0.5–17.9)	ns			
	BASO (%)	0.7 (0–1)	0.5 (0–3)	0.7 (0.4–1.2)	ns			
**Lipid profile**								
	Cholesterol (mg/dL)	196 (141–228)	242 (207–316)	162 (104–369)	<0.0001	<0.0001	0.0171	<0.0001
	Triglyceride (mg/dL)	159 (53–266)	169 (48–425)	125 (63–577)	ns			
	HDL (mg/dL)	50 (40–116)	54 (36–98)	54 (18–97)	ns			
	LDL (mg/dL)	101 (68–154)	138 (60–200)	84 (6–236)	<0.0001	0.0003	0.0277	<0.0001

**Note:**

All patients and controls were Thai postmenopausal women. N, normal; H, hyperlipidemia; and CHD, patients diagnosed with coronary heart disease. Data are shown as median (minimum-maximum). The comparison of values was determined by **Mann–Withney test and *Kruskal–Wallis test. The α level was set at <0.05 with a 95% confidence interval. WBC, white blood cell; RBC, red blood cells; Hb, hemoglobin, HCT, hematocrit; PLT, platelet; NEUT, neutrophil; LYMPH, lymphocyte; MONO, monocyte; EO, eosinophil; BASO, basophil; HDL, high density lipoprotein; LDL, low density lipoprotein.

### mRNA expression in PBMC extracts

**Figure 2:**
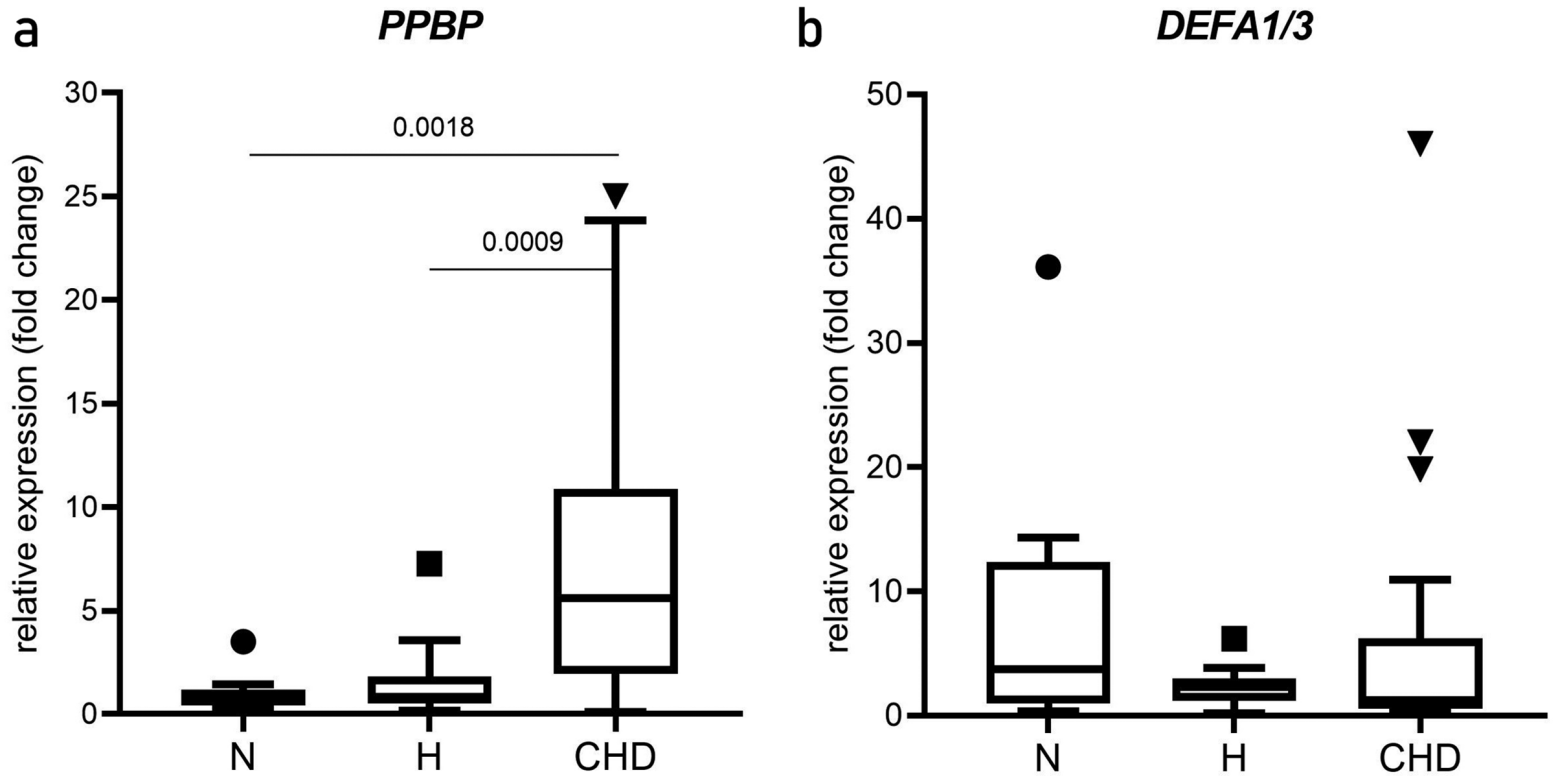
Expression of *PPBP* and *DEFA1/DEFA3* genes. Box and whisker plots of *PPBP* (A) and *DEFA1/DEFA3* gene expressions (B) showing altered expression in patient groups *vs*. controls. mRNA expression (2.0-fold change) relative to β-actin mRNA in PBMCs obtained from normal, and hyperlipidemia (H) and patients who were diagnosed coronary heart disease (CHD), as determined by RT-qPCR. Horizontal bar, box edges, and whiskers represent the median, the first/third quartiles, and the min/max values, respectively. The comparison of values between groups was determined by Mann-Whitney test. The α level was set at <0.05 with a 95% confidence interval.

The relative mRNA expression levels (mean two-fold changes) of *PPBP* and *DEFA1/DEFA3* in the N, H, and CHD groups are shown in Fig. 2. *PPBP* mRNA levels were significantly higher in the CHD group (5.439, 0.173–25.02) compared with the N (0.8470, 0.265–3.506), *p* = 0.0018) and H groups (0.173, 0.830–7.260), *p* = 0.0009) (Fig. 2A). *DEFA1/DEFA3* mRNA levels were not significantly different among N, H, and CHD groups (*p* > 0.05) (Fig. 2B).

### Plasma levels of PPBP and HNP-1–3

**Figure 3:**
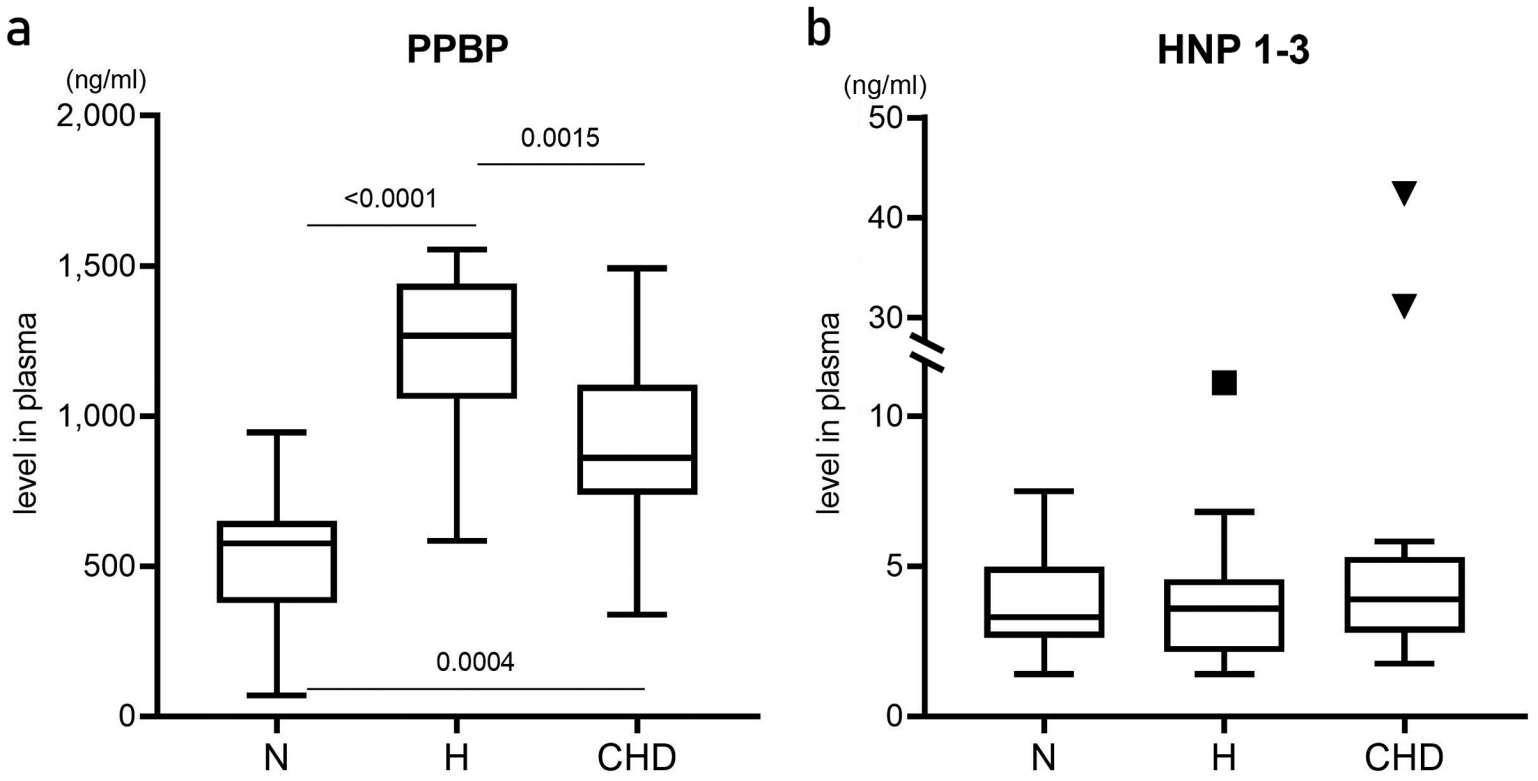
Plasma levels of pro-platelet basic protein, and human neutrophil peptides 1–3. Box and whisker plots showing plasma levels (ng/ml) of pro-platelet basic protein (PPBP) (A), and human neutrophil peptides 1–3 (HNP 1–3) (B) from healthy normal control (N) (*n* = 12), hyperlipidemia patients (H) (*n* = 18), and patients who were diagnosed coronary heart disease (CHD) (*n* = 21). Horizontal bar, box edges, and whiskers represent the median, the first/third quartiles, and the min/max values, respectively. The comparison of values between groups was determined by Mann–Whitney test. The α level was set at <0.05 with a 95% confidence interval.

Plasma levels of PPBP and HNP-1–3 proteins were determined by ELISA and compared among the N, H, and CHD groups (Fig. 3). PPBP levels were significantly higher in the H (1268.23, 583.579–1154.980 ng/ml, *p* < 0.0001) and CHD (867.965, 556.912–1492.70 ng/ml, *p* = 0.0004) groups compared with the N group (575.685, 68.842–944.632 ng/ml) (Fig. 3a). In contrast, there were no significant differences in HNP-1–3 levels among the patient and control groups (*p* > 0.05) (Fig. 3b).

### Correlations between mRNA expression, plasma protein levels, and clinical manifestations

The correlations among characteristics/clinical manifestations, *PPBP* and *DEFA1/DEFA3* mRNA expression, and plasma PPBP and HNP-1–3 levels are shown in Table S2. Plasma PPBP levels were significantly correlated with age (*r* = 0.3950, *p* = 0.0307), TC (*r* = 0.5794, *p* = 0.0008), LDL (*r* = 0.4923, *p* = 0.0057), and number of platelets (*r* = 0.4067, *p* = 0.0257). In contrast, plasma HNP-1–3 levels were not significantly associated with any clinical parameters. Neither *DEFA1/DEFA3* mRNA nor plasma levels of HNP-1–3 were correlated with number of neutrophils (*p* > 0.05).

## Discussion

We recently reported that the *PPBP* and *DEFA1-DEFA3* genes and their corresponding proteins, PPBP and HNP-1–3, were potential biomarkers for CHD risk in hyperlipidemic Thai men (Maneerat et al., 2016, 2017). The present study aimed to prove if these markers might also be used to predict CHD development in hyperlipidemic postmenopausal Thai women. We conducted a cross-sectional observational study in postmenopausal Thai women, to represent the spectrum of development from normolipidemia to hyperlipidemia and finally CHD. All participants had low estrogen levels, as an important CHD risk factor (Anand et al., 2008; Garcia et al., 2016; Nie et al., 2022) The current findings and knowledge of platelets and their protein functions (Gleissner, von Hundelshausen & Ley, 2008; Junt et al., 2007; Meadows & Bhatt, 2007; Nording, Seizer & Langer, 2015) suggested that *PPBP* and its protein were potential predictive biomarkers for CHD risk in postmenopausal Thai women. In contrast however, in this study we found that the *DEFA1/DEFA3* genes and encoded protein were not suitable markers.

The current findings revealed that *PPBP* mRNA expression levels were significantly increased in the CHD and H groups compared with the N group. In addition, plasma levels of PPBP protein were associated with lipid profiles. These findings, together with our previous results, suggested that PPBP mRNA and protein may be appropriate markers for CHD risk in both males and females (Maneerat et al., 2017). PPBP is known as a platelet-rich marker because it is a major protein component in activated platelets (Eicher et al., 2016; Stankiewicz et al., 2014). Similar to other vascular diseases, PPBP was recently reported as a potential biomarker for predicting acute ischemic stroke due to large vessel occlusion (Moin et al., 2021; Qin et al., 2019) and calcific aortic valve disease (Qiao, Huang & Wang, 2022). Moreover, PPBP has also been identified as a biomarker for other diseases, such as cancers (*e.g*. gastric cancer (Chen et al., 2022) and thyroid carcinoma (Zhang et al., 2021b)), inflammation (*e.g*. rheumatoid arthritis (Guerrero et al., 2021)), neurosyphilis (Li et al., 2020), COVID-19 (Yatim et al., 2021), and metabolic disease (*e.g*. diabetes mellitus (Moin et al., 2021; Zhang et al., 2021a)).

The present results suggested that expression of *DEFA1/DEFA3* and the encoded proteins HNP-1–3 did not differ significantly among the groups. In addition, neutrophil numbers were similar in all groups, even though neutrophils are an important factor in all stages of atherogenesis correlated with CVD (Kawaguchi et al., 1996; Naruko et al., 2002; Ovbiagele et al., 2007; Quinn et al., 2008; Weber & Noels, 2011). Neutrophils act as initiators of innate immunity in atherogenesis, are emerging players in atherosclerosis and infiltrate and are involved in the formation of atherosclerotic plaques, and are chronic atherogenic triggers and present in advanced plaques (Weber & Noels, 2011). However, we confirmed that our results were reliable and not the result of a technical error, and the appropriate positive controls were used. It is possible that there are differences in neutrophil biology and functions between adult males and females related to sex hormones (Maianski et al., 2004). Previous studies suggested that estradiol plays a role in modulating neutrophil phenotypes by triggering granulocyte-macrophage stimulating factor production to regulate neutrophil maturation (Robertson, Mayrhofer & Seamark, 1996). Adult males have more neutrophils with more-effective functioning than adult females (Casimir et al., 2010). In contrast, neutrophils in prepubescent males and females with incomplete estrogen levels showed similar maturation statuses (Maianski et al., 2004). This could help to explain the observed lack of difference in *DEFA1/DEFA3* and HNP-1–3 expression levels among the N, H, and CHD groups. Low estrogen levels in postmenopausal women may thus be associated with decreased neutrophil activity (Casimir et al., 2010; Gupta et al., 2020; Maianski et al., 2004). Moreover, the sex differences in the plaque morphology of coronary atherosclerosis are reported but still incompletely understood. The frequency of plaque erosion is higher in young women who smoke. Whereas plaques rupture is found higher in older women as compared to younger, in women and men with hyperlipidemia. The incidence and degree of coronary calcification are also different by sex. Less calcification is found in premenopausal women. It is necessary to elucidate these finding to better manage CVD (Sato et al., 2022).

The current study had some limitations, including (1) our sample size was small and did not meet the requirement of minimum sample size. Statistical bias might occur. Small sample size could affect the findings, more patient recruitment in each group would strengthen the study. (2) the gene profile was obtained and applied from male samples using next-generation sequencing analysis (Maneerat et al., 2016, 2017). The data might thus not be applicable in women, because of sex differences, for instance in innate immunity (Casimir et al., 2010; Gupta et al., 2020; Maianski et al., 2004; Stubelius et al., 2017), various risk factors (Anand et al., 2008; Gonzalez-Jaramillo et al., 2022; Sucato et al., 2022), and plaque morphology in coronary atherosclerosis (Sato et al., 2022). Further studies with larger sample size are therefore needed to validate the findings using sex-specific gene profiles to select appropriate biomarkers for females.

## Conclusions

We recently reported that the *PPBP* and *DEFA1/DEFA3* genes could act as biomarkers for CHD risk in Thai men with hyperlipidemia. The results of this study suggest that gene and protein levels of PPBP, but not *DEFA1/DEFA3*, and HNP-1–3, may also be feasible biomarkers for CHD risk in postmenopausal Thai women. Further multicenter studies with larger sample sizes are needed to confirm the current results and to identify more tools for predicting CHD risks in women worldwide.

## Supplemental Information

10.7717/peerj.13615/supp-1 Supplemental Information 1 General description and clinical manifestations of the study groups. All patients were Thai postmenopausal women. H = hyperlipidemia, and HD = patients with hyperlipidemia and had cholesterol lowering drug treatment. Data are shown as median (minimum-maximum). The comparison of valves between groups was determined by Mann-Whitney test. The α level was set at <0.05 with a 95% confidence interval. WBC =white blood cell, RBC = red blood cells, Hb =hemoglobin, HCT = hematocrit, PLT = platelet, NEUT = neutrophil, LYMPH = lymphocyte, MONO = monocyte, EO = eosinophil, BASO = basophil, HDL = high density lipoprotein, LDL = low density lipoprotein

10.7717/peerj.13615/supp-2 Supplemental Information 2 Correlation between clinical manifestations, *PPBP* and *DEFA1/DEFA3* mRNA expressions, and levels of plasma PPBP and HNP1-3. The correlation was analyzed by Spearman rank correlation test. The α level was set at <0.05 with a 95% confidence interval. *PPBP* = *PPBP* gene, *DEFA1/3 = DEFA1/DEFA3* genes, PPBP = pro-platelet basic protein, HNP 1-3 = human neutrophil peptides 1, 2, 3, WBC =white blood cell, RBC = red blood cells, Hb =hemoglobin, HCT = hematocrit, PLT = platelet, NEUT = neutrophil, LYMPH = lymphocyte, MONO = monocyte, EO = eosinophil, BASO = basophil, TC = total cholesterol, TG = triglyceride, HDL = high density lipoprotein, LDL = low density lipoprotein

10.7717/peerj.13615/supp-3 Supplemental Information 3 Raw data for Table 1, Figures 2 and 3.

## Additional Information and Declarations

### Data Availability

The following information was supplied regarding data availability: The raw data is available in the Supplemental File.
