# Supplementary material for: PPBP gene as a biomarker for coronary heart disease risk in postmenopausal Thai women
Source: PeerJ. 2022 Jun 17;10:e13615. doi: 10.7717/peerj.13615 (PMC9208370; doi:10.7717/peerj.13615)
Supplement: Supplemental Information 1 — All patients were Thai postmenopausal women. H = hyperlipidemia, and HD = patients with hyperlipidemia and had cholesterol lowering drug treatment. Data are shown as median (minimum-maximum). The comparison of valves between groups was determined by Mann-Whitney test. The α level was set at <0.05 with a 95% confidence interval. WBC =white blood cell, RBC = red blood cells, Hb =hemoglobin, HCT = hematocrit, PLT = platelet, NEUT = neutrophil, LYMPH = lymphocyte, MONO = monocyte, EO = eosinophil, BASO = basophil, HDL = high density lipoprotein, LDL = low density lipoprotein [file peerj-10-13615-s001.docx]

Supplementary Table 1:

General description and clinical manifestations of the study groups

| **Variable** | | **H** | | **HD** | |  | |  |
| --- | --- | --- | --- | --- | --- | --- | --- | --- |
|  |  | *(n=18)* | | *(n=39)* | | *p*-value | |  |
| Age (year) | | 56.5 (47-72) | | 58 (45-66) | | ns | |  |
| **Complete blood count** | | |  | |  | |  | |
|  | WBC (10^3^/uL) | 7 (4.1-8.1) | | 5.4 (2.5-9.2) | | 0.0146 | |  |
|  | RBC (10^3^/uL) | 4.4 (2.9-6.0) | | 4.7 (3.5-6.3) | | 0.0280 | |  |
|  | Hb (g/dL) | 12.8 (0.8-14.8) | | 13.8 (1-18) | | 0.0279 | |  |
|  | HCT (%) | 38.2 (25.4-44.6) | | 41.9 (31.1-54.3) | | 0.0004 | |  |
|  | PLT (10^3^/uL) | 289.5 (206-415) | | 254 (141-412) | | 0.0292 | |  |
|  | NEUT (%) | 52.4 (33-60.9) | | 56.6 (38-70) | | 0.0147 | |  |
|  | LYMPH (%) | 38.7 (3.2-54) | | 34.5 (17-50) | | ns | |  |
|  | MONO (%) | 5 (3.3-6.2) | | 5.5 (1-10) | | ns | |  |
|  | EO (%) | 2.1 (1-5) | | 2.5 (0.8-8.5) | | ns | |  |
|  | BASO (%) | 0.5 (0-3) | | 0.4 (0-2) | | ns | |  |
| **Lipid profile** | |  | |  | |  | |  |
|  | Cholesterol (mg/dL) | 242 (207-316) | | 187 (19-281) | | <0.0001 | |  |
|  | Triglyceride (mg/dL) | 169 (48-425) | | 114 (35-285) | | 0.0048 | |  |
|  | HDL (mg/dL) | 54 (36-98) | | 62.5 (4-101) | | ns | |  |
|  | LDL (mg/dL) | 138 (60-200) | | 99 (54-179) | | <0.0001 | |  |

All patients were Thai postmenopausal women.

H = hyperlipidemia, and HD = patients with hyperlipidemia and had cholesterol lowering drug treatment. Data are shown as median (minimum-maximum). The comparison of valves between groups was determined by Mann-Whitney test. The α level was set at <0.05 with a 95% confidence interval.

WBC =white blood cell, RBC = red blood cells, Hb =hemoglobin, HCT = hematocrit, PLT = platelet, NEUT = neutrophil, LYMPH = lymphocyte, MONO = monocyte, EO = eosinophil, BASO = basophil, HDL = high density lipoprotein, LDL = low density lipoprotein
