# Supplementary material for: PPBP gene as a biomarker for coronary heart disease risk in postmenopausal Thai women
Source: PeerJ. 2022 Jun 17;10:e13615. doi: 10.7717/peerj.13615 (PMC9208370; doi:10.7717/peerj.13615)
Supplement: Supplemental Information 2 — The correlation was analyzed by Spearman rank correlation test. The α level was set at <0.05 with a 95% confidence interval. PPBP = PPBP gene, DEFA1/3 = DEFA1/DEFA3 genes, PPBP = pro-platelet basic protein, HNP 1-3 = human neutrophil peptides 1, 2, 3, WBC =white blood cell, RBC = red blood cells, Hb =hemoglobin, HCT = hematocrit, PLT = platelet, NEUT = neutrophil, LYMPH = lymphocyte, MONO = monocyte, EO = eosinophil, BASO = basophil, TC = total cholesterol, TG = triglyceride, HDL = high density lipoprotein, LDL = low density lipoprotein [file peerj-10-13615-s002.docx]

Supplementary Table 2

Correlation between clinical manifestations, PPBP and DEFA1/DEFA3 mRNA expressions, and levels of plasma PPBP and HNP1-3 of N and H group.

|  | ***PPBP*** | **PPBP (ng/ml)** | **DEFA1/3** | **HNP1-3 (ng/ml)** | **Age** | **Cholesterol** | **Triglyceride** | **HDL** | **LDL** | **WBC**  **(10'3/uL)** | **RBC**  **(10'3/uL)** | **Hb**  **(g/dL)** | **HCT**  **(%)** | **PLT** | **NEUT** | **LYMPH**  **(%)** | **MONO**  **(%)** | **EO** | **BASO** |
| --- | --- | --- | --- | --- | --- | --- | --- | --- | --- | --- | --- | --- | --- | --- | --- | --- | --- | --- | --- |
| ***PPBP*** | 1.0000 | -0.1335 | 0.1141 | 0.2236 | -0.1054 | 0.1115 | 0.0160 | 0.06213 | 0.0906 | 0.0287 | 0.1693 | 0.1117 | 0.1164 | -0.0561 | 0.0670 | -0.0049 | -0.1845 | 0.1957 | -0.1127 |
|  |  | (0.4819) | (0.5481) | (0.2349) | (0.5792) | (0.5574) | (0.933) | (0.7443) | (0.634) | (0.8803) | (0.3711) | (0.5566) | (0.5402) | (0.7685) | (0.7251) | (0.9795) | (0.3290) | (0.3000) | (0.5532) |
| **PPBP**  **(ng/ml)** | -0.1335 | 1.0000 | -0.2360 | -0.0941 | **0.3950** | **0.5794** | 0.1428 | 0.0022 | **0.4923** | -0.0111 | 0.1613 | 0.2513 | 0.2141 | **0.4067** | 0.0078 | -0.0583 | -0.2415 | -0.2419 | -0.2200 |
|  | (0.4819) |  | (0.2092) | (0.6208) | **(0.0307)** | **(0.0008)** | (0.4514) | (0.9907) | **(0.0057)** | (0.9535) | (0.3945) | (0.1804) | (0.2560) | **(0.0257)** | (0.9674) | (0.7596) | (0.1985) | (0.1978) | (0.2428) |
| **DEFA1/3** | 0.1141 | -0.2360 | 1.0000 | 0.1698 | -0.1230 | -0.2898 | 0.2347 | -0.1416 | -0.1509 | 0.0156 | 0.0305 | 0.1362 | 0.1475 | -0.2396 | 0.1778 | -0.2942 | 0.1668 | 0.1437 | 0.0489 |
|  | (0.5481) | (0.2092) |  | (0.3698) | (0.5172) | (0.1203) | (0.2118) | (0.4554) | (0.4261) | (0.9349) | (0.8730) | (0.4729) | (0.4366) | (0.2022) | (0.3473) | (0.1146) | (0.3784) | (0.4488) | (0.7975) |
| **HNP1-3**  **(ng/ml)** | 0.2236 | -0.0941 | 0.1698 | 1.0000 | 0.1362 | -0.0870 | 0.0596 | **-0.3660** | 0.1943 | 0.2611 | 0.1577 | 0.0658 | 0.0957 | -0.0567 | **0.3885** | **-0.4689** | 0.0624 | 0.1357 | **-0.3685** |
|  | (0.2349) | (0.6208) | (0.3698) |  | (0.4728) | (0.6474) | (0.7543) | **(0.0467)** | (0.3035) | (0.1634) | (0.4051) | (0.7298) | (0.6149) | (0.7658) | **(0.0339)** | **(0.0090)** | (0.7432) | (0.4747) | **(0.0451)** |
| **Age** | -0.1054 | **0.3950** | -0.1230 | 0.1362 | 1.0000 | 0.0585 | 0.0458 | -0.2447 | 0.1549 | 0.1051 | 0.2133 | 0.2599 | 0.2536 | -0.1104 | -0.1947 | 0.0195 | -0.0751 | -0.3411 | 0.1255 |
|  | (0.5792) | **(0.0307)** | (0.5172) | (0.4728) |  | (0.7587) | (0.8100) | (0.1925) | (0.4138) | (0.5803) | (0.2577) | (0.1654) | (0.1763) | (0.5615) | (0.3024) | (0.9185) | (0.6933) | (0.0651) | (0.5086) |
| **Cholesterol** | 0.1115 | **0.5794** | -0.2898 | -0.0870 | 0.0585 | 1.0000 | 0.1799 | 0.3131 | **0.6802** | -0.0120 | 0.0855 | 0.0049 | -0.0502 | 0.1233 | -0.1946 | 0.2559 | -0.0803 | -0.1950 | -0.1691 |
|  | (0.5574) | **(0.0008)** | (0.1203) | (0.6474) | (0.7587) |  | (0.3415) | (0.0920) | **(<0.0001)** | (0.9497) | (0.6534) | (0.9795) | (0.7922) | (0.5162) | (0.3029) | (0.1722) | (0.6732) | (0.3018) | (0.3716) |
| **Triglyceride** | 0.0160 | 0.1428 | 0.2347 | 0.0596 | 0.0458 | 0.1799 | 1.0000 | **-0.5295** | 0.1424 | **0.5142** | 0.1600 | 0.1353 | 0.1005 | 0.3502 | -0.0590 | 0.0792 | -0.0295 | -0.1651 | -0.0341 |
|  | (0.933) | (0.4514) | (0.2118) | (0.7543) | (0.8100) | (0.3415) |  | **(0.0026)** | (0.4527) | **(0.0036)** | (0.3984) | (0.4758) | (0.5973) | (0.0578) | (0.7569) | (0.6773) | (0.8771) | (0.3834) | (0.8578) |
| **HDL** | 0.06213 | 0.0022 | -0.1416 | **-0.3660** | -0.2447 | 0.3131 | **-0.5295** | 1.0000 | -0.1172 | **-0.3752** | -0.1118 | -0.3358 | -0.2775 | -0.0867 | -0.2247 | 0.2621 | -0.0128 | -0.0225 | -0.1153 |
|  | (0.7443) | (0.9907) | (0.4554) | **(0.0467)** | (0.1925) | (0.0920) | **(0.0026)** |  | (0.5375) | **(0.0411)** | (0.5565) | (0.0696) | (0.1376) | (0.6486) | (0.2326) | (0.1617) | (0.9463) | (0.9060) | (0.5442) |
| **LDL** | 0.0906 | **0.4923** | -0.1509 | 0.1943 | 0.1549 | **0.6802** | 0.1424 | -0.1172 | 1.0000 | 0.1293 | 0.2121 | 0.2844 | 0.1868 | 0.0885 | 0.1035 | -0.0792 | -0.1214 | -0.1417 | -0.1711 |
|  | (0.634) | **(0.0057)** | (0.4261) | (0.3035) | (0.4138) | **(<0.0001)** | (0.4527) | ( 0.5375) |  | (0.4960) | (0.2605) | (0.1277) | (0.3231) | (0.6420) | (0.5863) | (0.6772) | (0.5229) | (0.4551) | (0.3659) |
| **WBC**  **(10'3/uL)** | 0.0287 | -0.0111 | 0.0156 | 0.2611 | 0.1051 | -0.0120 | **0.5142** | **-0.3752** | 0.1293 | 1.0000 | 0.1917 | -0.0204 | -0.0913 | 0.1488 | 0.1356 | -0.1385 | -0.0616 | 0.0893 | **-0.3748** |
|  | (0.8803) | (0.9535) | (0.9349) | (0.1634) | (0.5803) | (0.9497) | **(0.0036)** | **(0.0411)** | (0.4960) |  | (0.3103) | (0.9149) | (0.6314) | (0.4325) | (0.4750) | (0.4655) | (0.7462) | (0.6390) | **(0.0413)** |
| **RBC**  **(10'3/uL)** | 0.1693 | 0.1613 | 0.0305 | 0.1577 | 0.2133 | 0.0855 | 0.1600 | -0.1118 | 0.2121 | 0.1917 | 1.0000 | **0.4721** | **0.5581** | -0.0968 | 0.3019 | -0.2628 | **-0.3869** | 0.0056 | -0.1571 |
|  | (0.3711) | (0.3945) | (0.8730) | (0.4051) | (0.2577) | (0.6534) | (0.3984) | (0.5565) | (0.2605) | (0.3103) |  | **(0.0084)** | **(0.0014)** | (0.6109) | (0.1049) | (0.1606) | **(0.0347)** | (0.9767) | (0.4071) |
| **Hb (g/dL)** | 0.1117 | 0.2513 | 0.1362 | 0.0658 | 0.2599 | 0.0049 | 0.1353 | -0.3358 | 0.2844 | -0.0204 | **0.4721** | 1.0000 | **0.9362** | -0.1791 | 0.1192 | -0.1008 | -0.3004 | -0.1110 | 0.1180 |
|  | (0.5566) | (0.1804) | (0.4729) | (0.7298) | (0.1654) | (0.9795) | (0.4758) | (0.0696) | (0.1277) | (0.9149) | **(0.0084)** |  | **(<0.0001)** | (0.3437) | (0.5304) | (0.5959) | (0.1068) | (0.5593) | (0.5345) |
| **HCT (%)** | 0.1164 | 0.2141 | 0.1475 | 0.0957 | 0.2536 | -0.0502 | 0.1005 | -0.2775 | 0.1868 | -0.0913 | **0.5581** | **0.9362** | 1.0000 | -0.2090 | 0.1816 | -0.1640 | **-0.3848** | -0.1048 | 0.1019 |
|  | (0.5402) | (0.2560) | (0.4366) | (0.6149) | (0.1763) | (0.7922) | (0.5973) | (0.1376) | (0.3231) | (0.6314) | **(0.0014)** | **(<0.0001)** |  | (0.2678) | (0.3369) | (0.3864) | **(0.0357)** | (0.5814) | (0.5921) |
| **PLT** | -0.0561 | **0.4067** | -0.2396 | -0.0567 | -0.1104 | 0.1233 | 0.3502 | -0.0867 | 0.0885 | 0.1488 | -0.0968 | -0.1791 | -0.2090 | 1.0000 | 0.1285 | -0.1577 | 0.0645 | -0.0801 | -0.1607 |
|  | (0.7685) | **(0.0257)** | (0.2022) | (0.7658) | (0.5615) | (0.5162) | (0.0578) | (0.6486) | (0.6420) | (0.4325) | (0.6109) | (0.3437) | (0.2678) |  | (0.4986) | (0.4053) | (0.7348) | (0.6740) | (0.3962) |
| **NEUT** | 0.0670 | 0.0078 | 0.1778 | **0.3885** | -0.1947 | -0.1946 | -0.0590 | -0.2247 | 0.1035 | 0.1356 | 0.3019 | 0.1192 | 0.1816 | 0.1285 | 1.0000 | **-0.9342** | -0.1165 | 0.2670 | **-0.4910** |
|  | (0.7251) | (0.9674) | (0.3473) | **(0.0339)** | (0.3024) | (0.3029) | (0.7569) | (0.2326) | (0.5863) | (0.4750) | (0.1049) | (0.5304) | (0.3369) | (0.4986) |  | **(<0.0001)** | (0.5397) | (0.1538) | **(0.0059)** |
| **LYMPH (%)** | -0.0049 | -0.0583 | -0.2942 | **-0.4689** | 0.0195 | 0.2559 | 0.0792 | 0.2621 | -0.0792 | -0.1385 | -0.2628 | -0.1008 | -0.1640 | -0.1577 | **-0.9342** | 1.0000 | 0.0587 | -0.3082 | **0.4290** |
|  | (0.9795) | (0.7596) | (0.1146) | **(0.0090)** | (0.9185) | (0.1722) | (0.6773) | (0.1617) | (0.6772) | (0.4655) | (0.1606) | (0.5959) | (0.3864) | (0.4053) | **(<0.0001)** |  | (0.7579) | (0.0975) | **(0.0180)** |
| **MONO (%)** | -0.1845 | -0.2415 | 0.1668 | 0.0624 | -0.0751 | -0.0803 | -0.0295 | -0.0128 | -0.1214 | -0.0616 | -0.3869 | -0.3004 | **-0.3848** | 0.0645 | -0.1165 | 0.0587 | 1.0000 | 0.0910 | 0.0932 |
|  | (0.3290) | (0.1985) | (0.3784) | (0.7432) | (0.6933) | (0.6732) | (0.8771) | (0.9463) | (0.5229) | (0.7462) | (0.0347) | (0.1068) | **(0.0357)** | (0.7348) | (0.5397) | (0.7579) |  | (0.6326) | (0.6243) |
| **EO** | 0.1957 | -0.2419 | 0.1437 | 0.1357 | -0.3411 | -0.1950 | -0.1651 | -0.0225 | -0.1417 | 0.0893 | 0.0056 | -0.1110 | -0.1048 | -0.0801 | 0.2670 | -0.3082 | 0.0910 | 1.0000 | 0.0177 |
|  | (0.3000) | (0.1978) | (0.4488) | (0.4747) | (0.0651) | (0.3018) | (0.3834) | (0.9060) | (0.4551) | (0.6390) | (0.9767) | (0.5593) | (0.5814) | (0.6740) | (0.1538) | (0.0975) | (0.6326) |  | (0.9260) |
| **BASO** | -0.1127 | -0.2200 | 0.0489 | **-0.3685** | 0.1255 | -0.1691 | -0.0341 | -0.1153 | -0.1711 | **-0.3748** | -0.1571 | 0.1180 | 0.1019 | -0.1607 | **-0.4910** | **0.4290** | 0.0932 | 0.0177 | 1.0000 |
|  | (0.5532) | (0.2428) | (0.7975) | **(0.0451)** | (0.5086) | (0.3716) | (0.8578) | (0.5442) | (0.3659) | **(0.0413)** | (0.4071) | (0.5345) | (0.5921) | (0.3962) | **(0.0059)** | **(0.0180)** | (0.6243) | (0.9260) |  |

The correlation was analyzed by Spearman's correlation test. The α level was set at <0.05 with a 95% confidence interval.

*PPBP* = *PPBP* gene, *DEFA1/3 = DEFA1/DEFA3* genes, PPBP = pro-platelet basic protein, HNP 1-3 = human neutrophil peptides 1, 2, 3, WBC =white blood cell, RBC = red blood cells, Hb =hemoglobin, HCT = hematocrit, PLT = platelet, NEUT = neutrophil, LYMPH = lymphocyte, MONO = monocyte, EO = eosinophil, BASO = basophil, TC = total cholesterol, TG = triglyceride, HDL = high density lipoprotein, LDL = low density lipoprotein
